# *FGFRL1* and *FGF* genes are associated with height, hypertension, and osteoporosis

**DOI:** 10.1371/journal.pone.0273237

**Published:** 2022-08-18

**Authors:** Hye-Won Cho, Hyun-Seok Jin, Yong-Bin Eom

**Affiliations:** 1 Department of Medical Sciences, Graduate School, Soonchunhyang University, Asan, Chungnam, Republic of Korea; 2 Department of Biomedical Laboratory Science, College of Life and Health Sciences, Hoseo University, Asan, Chungnam, Republic of Korea; 3 Department of Biomedical Laboratory Science, College of Medical Sciences, Soonchunhyang University, Asan, Chungnam, Republic of Korea; Wright State University, UNITED STATES

## Abstract

Hypertension and osteoporosis are two major disorders, which interact with each other. Specific genetic signals involving the fibroblast growth factor receptor-like 1 (*FGFRL1*) gene are related to high blood pressure and bone growth in giraffes. *FGFRL1* is associated with cardiovascular system and bone formation. We performed an association study to investigate the role of *FGFRL1* in hypertension, osteoporosis, and height determination in humans. In addition, we identified three kinds of phenotypes in fibroblast growth factor (*FGF*) genes and examined their association with the *FGFRL1* gene. We identified 42 SNPs in the *FGFRL1* gene associated with each trait. We then analyzed the potential functional annotation of each SNP. The *FGFRL1* gene was found to be associated with height, hypertension, and osteoporosis, consistent with the results of a previous study. In addition, the *FGF2*, *FGF4*, *FGF10*, *FGF18*, and *FGF22* genes were found to interact with the *FGFRL1* gene. Our study suggests that both *FGFRL1* and *FGFRL1*-related genes may determine the height and the prevalence of osteoporosis and hypertension in the Korean population.

## Introduction

Osteoporosis is characterized by decreased bone mineral density and an increased risk of fractures, and is one of the most common chronic and metabolic bone diseases [1, 2]. Hypertension is the main risk factor for coronary heart disease, stroke, and chronic kidney disease, and is a prominent cause of death worldwide [3]. Both these diseases are metabolic conditions mediated via common pathophysiology, which means potential mechanistic links between osteoporosis and hypertension. Increasing evidence suggests that bone marrow-derived cells play a role in hypertension [4]. Osteoporosis and hypertension appear to be strongly triggered by immune cell activation, including enhanced salt intake, increased sympathetic outflow, and excessive angiotensin II and aldosterone [5]. Besides, various cohort studies reported the interplay between osteoporosis and hypertension [6–8].

A recent study identified specific genetic signals that are associated with adaptation to high blood pressure and bone growth in giraffes, with exceptional anatomy contributing to their unique stature and biological characteristics [9]. Accordingly, the strongest signal was located in the *FGFRL1* (fibroblast growth factor receptor-like 1) gene, and genetic experiments were performed in mice for further investigation. Mice carrying the giraffe-type *FGFRL1* allele showed resistance to hypertension and high bone mineral density, suggesting that the genetic changes induced pleiotropic effects related to cardiovascular development and bone formation [9].

*FGFRL1*, the most recently identified member of the *FGFR* (fibroblast growth factor receptor) family, is related to the cardiovascular system and bone formation [10–12]. Mutant FGFRL1 protein was found in a patient with craniosynostosis syndrome, indicating that the underlying gene was significantly associated with skeletal malformations [13]. Also, deletion of the *Fgfrl1* gene in a mouse model resulted in Wolf-Hirschhorn syndrome (WHS), including skeletal anomalies and congenital heart defects [10]. A few genome-wide association studies involving the *FGFRL1*gene were performed, and most of the studies analyzed bone mineral density [14, 15], while others investigated body height [14] and type 2 diabetes mellitus [16].

The present study is based not only on the correlation between hypertension and osteoporosis but also on previous studies, which elucidated the molecular mechanism of *FGFRL1*. We focused on the recent study, which demonstrated specific genetic signals related to high blood pressure and bone growth in giraffes [9, 17]. The previous study identified that the *FGFRL1* gene contributes to the representative biological characteristics of giraffes. Therefore, we confirmed the replications of the *FGFRL1* signals and identified that the *FGFRL1* gene is associated with giraffe-related characteristics (height, hypertension, and osteoporosis) in the human population. In addition, we analyzed gene-gene interaction networks. Expression and regulation levels of the *FGFRL1* gene were also analyzed in association with the three phenotypes, referring to the Genotype-Tissue Expression (GTEx) project. We identified the genetic variants of the *FGFRL1* gene as well as *FGFRL1*-related genes, which affect the skeletal system and/or hypertension.

## Materials and methods

### Study participants

The study data were obtained from the Korean Genome and Epidemiology Study [18] Health Examinees (HEXA) study [18]. The Korean Centers for Disease and Control (KCDC) recruited 173,357 participants for the HEXA study aged 40 to 79 years from 2004 to 2013 who lived in urban (Seoul, Incheon, Daejeon, Daegu, Ulsan, Busan, and Gwangju) and rural (Gyeonggi, Sejong, Gangwon, Chungcheongbuk, Chungcheongnam, Gyeonsangbuk, Gyeonsangnam, Jeollabuk, Jeollanam, and Jeju) areas of Korea [18]. Of these, only 58,698 participants who were available single nucleotide polymorphism (SNP) information and included in the baseline study were selected for the current study. The value of body measurements with each disease are shown in S1 Table. Height [19], weight [20], diastolic blood pressure (DBP), and systolic blood pressure (SBP) were measured. Height and weight were measured using an automated measuring instrument (Dong Sahn Jenix Co., Seoul, Korea) three times for the average values. Each diastolic and systolic blood pressure was measured three times every at intervals of more than 5 minutes by a mercury sphygmomanometer in a seated position, and the average value was used. Body mass index (BMI) was computed in units of kilograms per square meter (kg/m^2^), using the measured height and weight values. Patients with hypertension (n = 17,086) were defined by SBP ≥ 140 mmHg and/or DBP ≥ 90 mmHg, or a medical history of hypertension or use of antihypertensive medication. Controls (n = 31,440) were defined by SBP < 120 mmHg and DBP < 80 mmHg and no medical history of hypertension or anti-hypertensive drug use. The diagnosis of osteoporosis was made by a medical doctor based on bone indices measured via whole-body dual-energy X-ray absorptiometry (DXA). The final binary variable, the value we used in this study, was derived from the participants’ medical records through a questionnaire about whether there was a history of the previous diagnosis by a medical doctor. In total, after excluding 89 patients who were either non-respondents or had never been screened for osteoporosis, 55,535 controls and 3,074 cases of osteoporosis were identified.

### Genotyping

Genotype data were obtained by the Center for Genome Science, Korea National Institute of Health (KNIH). DNA samples were extracted from peripheral blood and genotyping was done using the Axiom^®^ 2.0 Reagent Kit (Affymetrix Axiom^®^ 2.0 Assay User Guide). Genotype data were generated using the KoreanChip (KCHIP) designed by the Center for Genome Science at the KNIH. Gene location was determined in reference to the National Center for Biotechnology Information (NCBI) Human Genome Build 37 (hg19) assembly. All the gene regions analyzed in this study were expanded by 5 kb at both transcripts ends, and SNPs were selected in this range. The KCHIP has been described comprehensively in previous studies [21]. The only genotypes that satisfied these exclusion criteria: low call rate (< 0.95%), sex inconsistency, cryptic first-degree relatives, and excessive heterozygosity. SNPs with genotype call rates < 95%, Hardy–Weinberg equilibrium (HWE) *p*-value < 10^−6^, and minor allele frequency of < 1% were removed. A total of 465,000 variants were included after quality control. A total of 8,056,211 SNPs were used for GWAS after quality control and imputation.

### Statistical analysis

PLINK version 1.90 beta (https://www.cog-genomics.org/plink2) was used for most statistical analyses. Imputation for autosomal variants was executed using IMPUTE2 with the reference panel constructed from 1000 phase 3 genomes. A logistic regression, additive genetic model was used after adjustments for age, sex, and body mass index (BMI) to investigate the association between SNPs in the *FGFRL1* gene and hypertension and osteoporosis. SNPs associated with height were identified via linear regression additive analysis adjusting for age and sex, with a cut-off *p*-value of *P* < 0.05. We sorted out tag SNP, the representative SNP in each genome region with high linkage disequilibrium (LD), from the haplotype block under the condition that the LD measure *r*^2^ ≥ 0.8. Regional association plots were generated using LocusZoom (http://locuszoom.org/). The HaploReg database (https://pubs.broadinstitute.org) was used to identify functional effects, such as protein motifs, in the *FGFRL1* genetic variants associated with both hypertension and osteoporosis. GTExPortal databases (https://gtexportal.org) were used for expression quantitative trait loci (eQTL) analysis. RegulomeDB (https://regulomedb.org/regulome-search/) was used to rank potential functional roles. We depicted annotated gene networks using STRING database version 11.0 (https://string-db.org/) and selected the genes with direct interactions with the *FGFRL1* gene.

### Ethical review

This study was approved by the Institutional Review Board of the Korean National Institute of Health (KNIH, KBN-2021-003) and Soonchunhyang University (202012-BR-086-01). Written informed consent was obtained from all participants.

## Results

### Association of the *FGFRL1* gene variants with height, hypertension, and osteoporosis

A total of 44 tag SNPs were identified in the *FGFRL1* gene. Logistic regression analysis for hypertension and osteoporosis using the additive genetic model revealed three and six nominally significant SNPs (*P* < 0.05), respectively (Table 1). Linear regression analysis for height identified nine significant SNPs. Among nine SNPs, 3 and 6 SNPs had positive and negative associations with height, respectively (Table 1). Two SNPs (rs13143527 and rs55639339) were associated with both hypertension and osteoporosis, but none of the height-related SNPs was related to osteoporosis or hypertension. Rs55639339 showed similar patterns of increased risk for hypertension (OR: 1.043) and osteoporosis (OR: 1.103), whereas the rs13143527 variant was associated with a decreased risk of hypertension (OR: 0.967) and osteoporosis (OR: 0.939) (Table 1). A regional plot of the *FGFRL1* gene based on height, hypertension, and osteoporosis was drawn using LocusZoom (S1 Fig).

**Table 1:** Genetic variants in *FGFRL1* associated with height, hypertension and osteoporosis.

SNP	Minor allele	MAF	Function	Height		HTN		Osteoporosis	
				β ± s.e	*p*-value	OR (95% CI)	*p*-value	OR (95% CI)	*p*-value
rs117864192	A	0.031	intron	-0.249 ± 0.087	**4.17 × 10** ^**−3**^	1.013 (0.935–1.098)	0.749	0.966 (0.829–1.126)	0.658
rs73219733	T	0.029	intron	-0.251 ± 0.091	**5.83 × 10** ^**−3**^	1.008 (0.926–1.097)	0.855	0.999 (0.849–1.175)	0.987
rs748650	A	0.255	intron	-0.090 ± 0.035	**9.77 × 10** ^**−3**^	1.017 (0.984–1.050)	0.315	1.047 (0.984–1.113)	0.146
rs115259783	G	0.157	intron	0.105 ± 0.041	**0.011**	0.979 (0.942–1.018)	0.287	1.031 (0.958–1.109)	0.421
rs34627176	A	0.044	upstream	-0.177 ± 0.074	**0.017**	0.967 (0.903–1.036)	0.345	0.959 (0.839–1.096)	0.537
rs139932728	A	0.021	upstream	0.248 ± 0.104	**0.017**	1.005 (0.913–1.106)	0.924	1.115 (0.931–1.335)	0.237
rs3733350	T	0.101	3’-UTR	0.113 ± 0.050	**0.025**	0.959 (0.915–1.005)	0.078	1.047 (0.959–1.143)	0.310
rs4647936	T	0.030	3’-UTR	-0.192 ± 0.089	**0.030**	1.053 (0.970–1.143)	0.215	0.928 (0.791–1.088)	0.358
rs77488513	T	0.030	upstream	-0.180 ± 0.088	**0.042**	1.059 (0.976–1.149)	0.170	0.917 (0.781–1.076)	0.286
**rs13143527 **	G	0.291	intron	-0.063 ± 0.033	0.057	0.967 (0.937–0.997)	**0.031**	0.939 (0.885–0.997)	**0.039**
**rs55639339**	T	0.140	intron	-0.015 ± 0.044	0.736	1.043 (1.002–1.086)	**0.041**	1.103 (1.023–1.190)	**0.011**
rs10010999	T	0.348	upstream	-0.028 ± 0.032	0.370	0.971 (0.942–0.999)	**0.047**	0.977 (0.924–1.034)	0.418
rs74921869	A	0.253	intron	0.033 ± 0.035	0.343	1.015 (0.983–1.048)	0.362	1.093 (1.028–1.161)	**4.35 × 10** ^**−3**^
rs35220088	C	0.309	intron	-0.003 ± 0.033	0.924	1.001 (0.972–1.032)	0.926	1.084 (1.024–1.148)	**5.86 × 10** ^**−3**^
rs73070422	G	0.140	intron	-0.015 ± 0.044	0.723	1.041 (0.999–1.083)	0.053	1.087 (1.008–1.173)	**0.031**
rs78590462	T	0.066	intron	-0.065 ± 0.061	0.287	1.019 (0.963–1.079)	0.505	0.892 (0.798–0.997)	**0.044**

Age, sex and body mass index (BMI) were included as covariants in all genetic models. SNPs indicated in bold are associated with both hypertension and osteoporosis at *P* < 0.05. Abbreviations: SNP, single nucleotide polymorphism; Chr, chromosome; MAF, minor allele frequency; HTN, hypertension; β, regression coefficient; s.e, standard error; OR, odds ratio; CI, confidence interval.

### Association of *FGF* genes with height, hypertension, and osteoporosis

**Fig 1:**
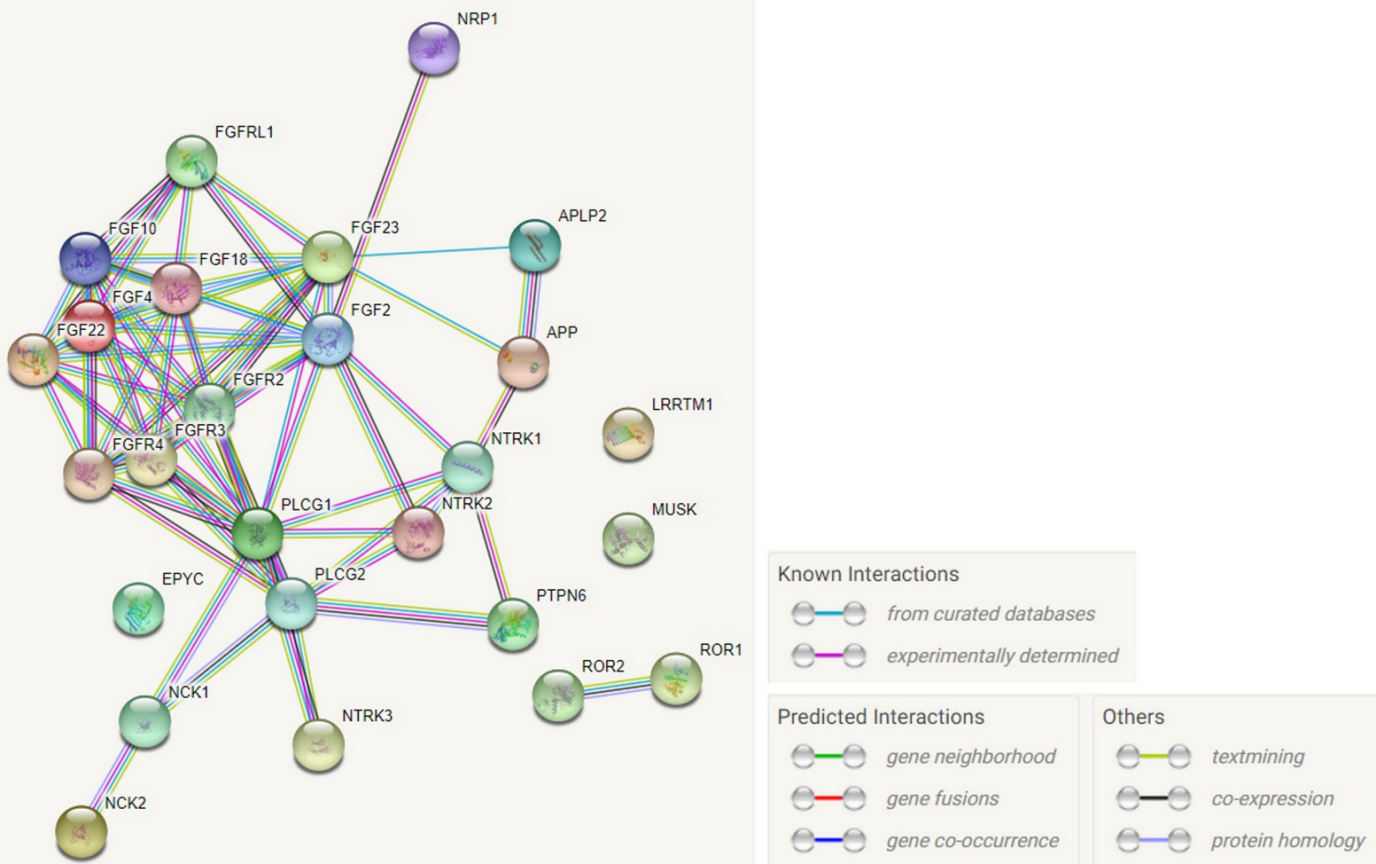
Protein-protein interactions with high confidence score (confidence score > 0.7) for *FGFRL1*. Proteins in the interaction network are represented as nodes; the line color represents the type of interaction, including known interaction, predicted interaction and other types. These interactions include physical (direct) and functional (indirect) types according to computational predictions and experimental repositories.

The gene-gene interaction networks generated by STRING revealed connections with high confidence scores (confidence score > 0.7) between *FGFRL1* and five fibroblast growth factors (*FGF2*, *FGF4*, *FGF10*, *FGF18*, and *FGF22*) (Fig 1). The *FGF10*, *FGF18*, *FGF2*, *FGF4*, and *FGF22* genes, which interact with *FGFRL1*, were correlated with height, hypertension, and osteoporosis. Overall, SNPs related to height were the most common, and no genetic variant of the *FGF4* gene was related to hypertension (S2 Table). Previous studies demonstrated an association between the *FGF18* gene and height [14, 24]. Interestingly, rs8109113 in the *FGF22* gene was associated with both hypertension and height, and 17 SNPs in *FGF10*, *FGF18*, and *FGF2* were associated with both osteoporosis and height (Table 2).

**Table 2:** Genetic variants in the *FGF* family associated with two of the three phenotypic traits (height, hypertension, and osteoporosis) or below the Bonferroni-corrected significance level.

Gene	Chr	SNP	Minor allele	MAF	Function	Height		HTN		Osteoporosis	
						β ± s.e	*p*-value	OR (95% CI)	*p*-value	OR (95% CI)	*p*-value
*FGF2*	4	rs167428	C	0.087	intron	0.112 ± 0.054	**0.037**	1.040 (0.99–1.09)	0.122	1.131 (1.03–1.24)	**8.59 × 10** ^**−3**^
	4	rs308387	A	0.052	intron	0.234 ± 0.068	**5.85 × 10** ^ **−4** ^	1.023 (0.96–1.09)	0.487	1.062 (0.94–1.20)	0.317
*FGF4*	11	rs58166091	A	0.213	-	0.114 ± 0.037	**2.16 × 10** ^ **−3** ^	0.989 (0.96–1.02)	0.544	1.026 (0.96–1.10)	0.436
*FGF10*	5	rs17227836	C	0.054	intron	-0.161 ± 0.067	**0.016**	0.993 (0.93–1.06)	0.816	1.144 (1.02–1.28)	**0.021**
	5	rs13154419	G	0.412	intron	0.112 ± 0.031	**2.89 × 10** ^ **−4** ^	0.991 (0.96–1.02)	0.545	0.988 (0.94–1.04)	0.674
	5	rs1448039	A	0.500	intron	-0.094 ± 0.030	**1.90 × 10** ^ **−3** ^	1.006 (0.98–1.04)	0.653	1.017 (0.96–1.07)	0.528
*FGF18*	5	rs10463007	T	0.404	-	0.081 ± 0.031	**9.26 × 10** ^**−3**^	0.990 (0.96–1.02)	0.498	1.071 (1.01–1.13)	**0.014**
*FGF22*	19	rs8109113	G	0.024	intron	-0.241 ± 0.100	**0.016**	0.901(0.82–0.99)	**0.028**	1.021 (0.85–1.22)	0.824

Age, sex and body mass index (BMI) were included as covariant in all genetic models. Findings with *P* < 0.05 are indicated in bold. The *p*-values which satisfied the Bonferroni-corrected significance level regarding each gene are indicated in bold and underlined. Abbreviations: SNP, single nucleotide polymorphism; Chr, chromosome; MAF, minor allele frequency; HTN, hypertension; β, regression coefficient; s.e, standard error; OR, odds ratio; CI, confidence interval.

### Functional analysis

**Fig 2:**
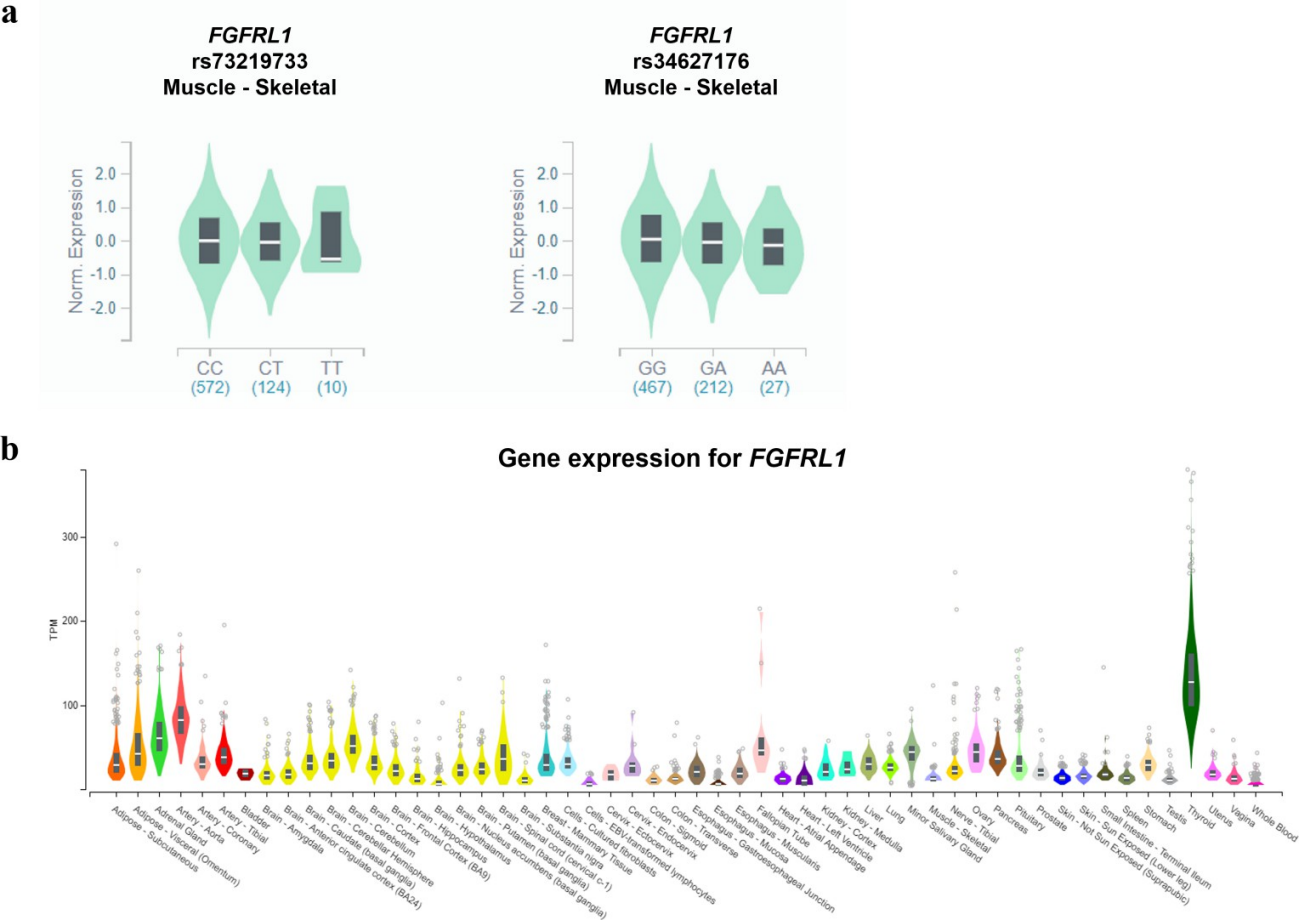
*FGFRL1* gene expression and identification of three SNPs in eQTL. Each genotype in skeletal muscle is expressed using GTExPortal. **(a)** Expression of rs73219733 and rs34627176 in *FGFRL1*. The two SNPs showed lower expression in their minor allele with statistical significance reaching *P* < 10^−4^. **(b)** Expression of the *FGFRL1* gene.

We used the GTEx database and HaploReg to determine the biological functional annotations of the identified genetic variants and genes (Fig 2). The GTEx database was used to obtain tissue expression data for *FGFRL1*. The *FGFRL1* gene is expressed in various tissues, especially in the thyroid gland and arteries (Fig 2B). Rs73219733 and rs34627176, which were associated with height, were significant signals of eQTL for the *FGFRL1* gene in skeletal muscle tissue (*P* = 8.3 × 10^−5^, 1.7 × 10^−12^) (Fig 2A). In addition, motif changes were predicted for the two SNPs that were significantly correlated with hypertension and osteoporosis (S3 Table). Based on the results, the *FGFRL1* gene expression varies with the SNP genotype.

## Discussion

Our study validated the correlation between *FGFRL1* gene and height, hypertension, and osteoporosis. The results are consistent with a previous study that suggested an association of *FGFRL1* with the cardiovascular and skeletal systems [10, 12]. We identified 16 SNPs in the *FGFRL1* gene that were associated with the traits of interest. We then annotated the potential biological function of these SNPs. Similarly, the *FGF2*, *FGF4*, *FGF10*, *FGF18*, and *FGF22* genes, which showed interactions with the *FGFRL1* gene, were associated with height, hypertension, and osteoporosis.

Most previous genetic association studies analyzed the association of *FGFRL1* with bone formation or bone density, and seldom with hypertension. In the present study, two SNPs were identified with significant (*P* < 0.05) association with both osteoporosis and hypertension: the minor allele of rs55639339 was associated with an increased risk of both diseases, while the minor allele of rs10010999 lowered the risk of both diseases, indicating a similar trend in prevalence of both diseases involving each genetic variant. Thus, our results are consistent with previous findings suggesting that osteoporosis is associated with metabolic diseases including hypertension [22]. Meanwhile, *FGFRL1* is known to be essential for vasculogenesis and ventricular septation. Indeed, *Fgfrl1* -/- embryos manifested defects in ventricular septation and congenital heart defects [10]. Additionally, a functional study in zebrafish revealed a significant role of *Fgfrl1* during gill cartilage development and craniofacial skeletogenesis [23–25]. Accordingly, the results of this replication study, which indicate that the genetic differences influence the risk of osteoporosis and hypertension, provide insight into the role of the *FGFRL1* gene while supporting previous functional studies.

It is well documented that the *FGF/FGFR* signaling axis plays an essential role in development, tissue homeostasis, and metabolism [26, 27]. Mutations and SNPs of *FGF*s are known associated with multiple skeletal disorders [14]. For instance, a constitutionally increased expression of the *FGF4* gene is a risk factor for craniosynostosis [27]. Overexpression of *FGF2* in mice resulted in shortened long bones along with defective mineralization and osteopenia, suggesting that the gene acts as a negative regulator of bone formation [28, 29]. Also, the genetic knockdown of *FGF10* is related to skeletal phenotypes such as craniosynostosis in a mouse model [30], and *FGF18* is expressed during osteoblast differentiation [31]. Indeed, the present study revealed significant genetic signals in *FGF2*, *FGF10*, and *FGF18* associated with height (S4 Table). Furthermore, several SNPs in *FGF18* were associated with osteoporosis (S2 Table).

By contrast, genetic variants in *FGF2* also showed an association with hypertension. *FGF2* is widely expressed in the myocardium, coronary vessels, and smooth muscle cells of the aorta [32, 33]. Besides, *FGF2* knock-out mice exhibit reduced vascular tension and decreased arterial blood pressure, suggesting autonomic dysfunction [34, 35]. Interestingly, all of the SNPs in *FGF2* that were associated with hypertension suggested that the risk of hypertension was diminished with minor alleles (S2 Table). These results presented that the *FGFRL1* signaling pathway could be considered in cardiovascular disease and hypertension in humans, suggesting that the variations in the *FGF2* gene implicate the heart or vascular tone.

Due to the absence of the intracellular tyrosine kinase domain in *FGFRL1*, which is essential for downstream *FGF* signaling, *FGFRL1* is widely assumed to act as a decoy receptor (competitive inhibitor) that joins and regulates *FGF* ligands [11, 36, 37]. Interaction between *FGF* and *FGFRs* is known to mediate several developmental phenomena, such as differentiation of mesenchymal stromal cells (MSCs) [38–40]. Kahkonen *et al*. showed that *FGFRL1* mRNA expression is remarkably increased during differentiation of MSCs into osteoblasts and adipocyte differentiation, and silencing of *FGFR1* and *FGFR2* in MSCs decreased the *FGFRL1* expression in osteoblasts and adipocytes [20]. The discovery that *FGFR1* and *FGFR2* modulate *FGFRL1* expression in MSCs highlighted the role of *FGFRL1* in MSC differentiation into osteoblasts and adipocytes [20]. Thus, consistent with studies associating genetic signals of the *FGFRL1* gene, *FGFR*, and *FGF* families, our results (gene-gene interaction network (Fig 1) and association test (Tables 1 and 2)) suggested that the interaction between *FGFRL1* and *FGF* family affects the cardiovascular and skeletal systems.

However, our study has several limitations. First, except for hypertension, none of the continuous variables (distal radius speed of sound (DR-SOS), midshaft tibia speed of sound (MT-SOS), and *T* score) related to osteoporosis were identified only depending on the medical history. Second, although lifestyle and comorbidities definitely influence the risk of hypertension and osteoporosis, we did not evaluate them in the present study. Further studies are needed to evaluate additional factors including gender, lifestyle, and co-morbid conditions.

In conclusion, we have replicated the association between *FGFRL1* and height, hypertension, and osteoporosis in the Korean population, and found genetic variants associated with each trait. The genetic variants in the *FGF* family members that interact with *FGFRL1*were associated with height, hypertension, and osteoporosis. The findings suggest that the giraffe-specific *FGFRL1* gene, which is associated with biological characteristics in giraffes, including tall stature and cardiovascular adaptations, is related to the corresponding phenotype in humans. Thus, our study provides an approach to the genetic basis of the pleiotropic effect of *FGF/FGFR* signaling.

## Supporting information

S1 Fig Regional association plot of the *FGFRL1* genetic variants. Signals related to (a) height, (b) hypertension and (c) osteoporosis in the *FGFRL1* gene are plotted as -log_10_ *P*-values. The color of each SNP plot shows its linkage disequilibrium (LD) (using *r*^2^ values) with the novel SNP (purple diamond) within the association locus. The y-axis on the right shows the recombination rate according to the HapMap database. The above image was constructed using the LocusZoom program (http://locuszoom.org/).
(TIF)

S1 Table Characteristics of the subjects in the HEXA study. (DOCX)

S2 Table Genetic variants in *FGF* family members associated with height, hypertension and osteoporosis. (DOCX)

S3 Table HaploReg results of genetic variants in *FGFRL1* that were associated with both hypertension and osteoporosis. Abbreviations: A1, minor allele; A2, major allele. The lower the Regulome DB score, the greater the effect on SNP.
(DOCX)

S4 Table Association of all genetic variants in *FGFRL1* and *FGF* family members with height, hypertension, and osteoporosis. Age, sex and body mass index (BMI) were included as covariant in all genetic models. SNPs associated with both hypertension and osteoporosis in common and had *P* < 0.05 are indicated in bold. The *p*-values which are satisfied the Bonferroni-corrected significance level regarding each gene are indicated in bold and underlined. Abbreviations: SNP, single nucleotide polymorphism; Chr, chromosome; MAF, minor allele frequency; HTN, hypertension; β, regression coefficient; s.e, standard error; OR, odd ratio; CI, confidence interval.
(XLSX)
