# Supplementary figures and images for: FGFRL1 and FGF genes are associated with height, hypertension, and osteoporosis
Source: PLoS One. 2022 Aug 18;17(8):e0273237. doi: 10.1371/journal.pone.0273237 (PMC9387819; doi:10.1371/journal.pone.0273237)

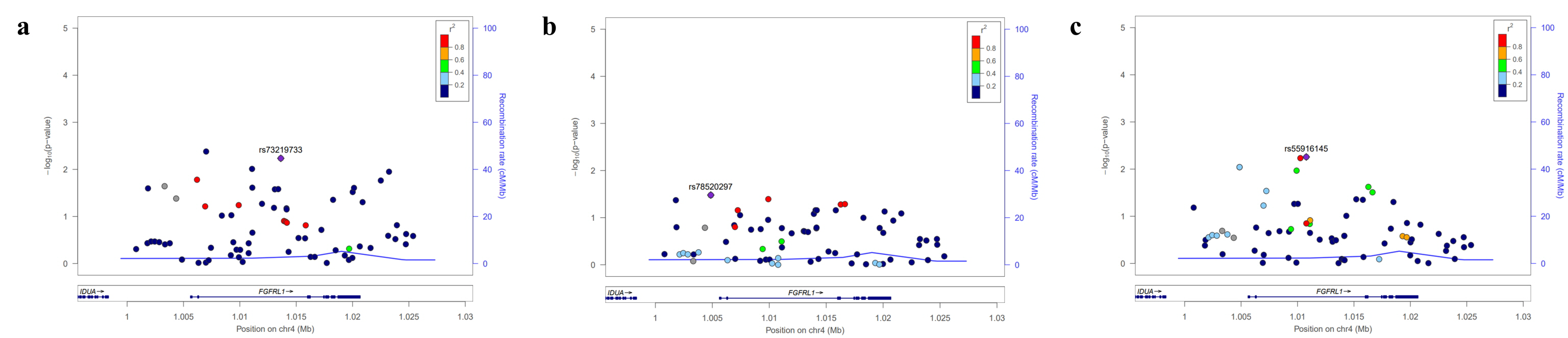

Supplement: S1 Fig — Signals related to (a) height, (b) hypertension and (c) osteoporosis in the FGFRL1 gene are plotted as -log10 P-values. The color of each SNP plot shows its linkage disequilibrium (LD) (using r2 values) with the novel SNP (purple diamond) within the association locus. The y-axis on the right shows the recombination rate according to the HapMap database. The above image was constructed using the LocusZoom program (http://locuszoom.org/). (TIF) [file pone.0273237.s001.tif]
