# Supplementary material for: FGFRL1 and FGF genes are associated with height, hypertension, and osteoporosis
Source: PLoS One. 2022 Aug 18;17(8):e0273237. doi: 10.1371/journal.pone.0273237 (PMC9387819; doi:10.1371/journal.pone.0273237)
Supplement: S1 Table — (DOCX) [file pone.0273237.s002.docx]

**S1 Table** Characteristics of the subjects in the HEXA study.

|  | Quantitative trait analysis | Hypertension | | | Osteoporosis | | | |
| --- | --- | --- | --- | --- | --- | --- | --- | --- |
|  |  | Case | Control | *P* value* | | Case | Control | *P* value* |
| Number of subjects | 58,698 | 17,086 | 31,440 |  | | 3,074 | 55,535 |  |
| Age (M years ± SD) | 53.80 ± 8.02 | 57.36 ± 7.45 | 51.97 ± 7.78 | <0.001 | | 59.58 ± 6.41 | 53.48 ± 7.97 | <0.001 |
| Sex (men (%)) | 20,293 (34.57%) | 7,430 (43.49%) | 8,589 (27.32%) | <0.001 | | 133 (4.33%) | 20,215 (36.40%) | <0.001 |
| Height (cm ± SD) | 160.72 ± 7.92 | 161.00 ± 8.31 | 160.22 ± 7.57 | <0.001 | | 155.26 ± 5.83 | 161.03 ± 7.92 | <0.001 |
| Weight (cm ± SD) | 61.89 ± 9.89 | 65.05 ± 10.32 | 59.71 ± 9.07 | <0.001 | | 56.63 ± 7.60 | 62.18 ± 9.92 | <0.001 |
| BMI (M kg/m² ± SD) | 23.88 ± 2.88 | 25.02 ± 2.95 | 23.20 ± 2.67 | <0.001 | | 23.49 ± 2.84 | 23.91 ± 2.88 | <0.001 |
| SBP (mmHg) | 122.46 ± 46 | 135.01 ± 14.63 | 113.85 ± 10.50 | <0.001 | | 123.34 ± 14.82 | 122.40 ± 14.78 | <0.001 |
| DBP (mmHg) | 75.76 ± 9.73 | 83.12 ± 9.90 | 69.72 ± 6.48 | <0.001 | | 74.95 ± 9.34 | 75.81 ± 9.75 | <0.001 |

Abbreviations: BMI, body mass index; M, mean value; SBP, systolic blood pressure; DBP, SD, standard deviation. *Significant differences in case and control groups were obtained by the Student's *t* test.
