# Supplementary material for: FGFRL1 and FGF genes are associated with height, hypertension, and osteoporosis
Source: PLoS One. 2022 Aug 18;17(8):e0273237. doi: 10.1371/journal.pone.0273237 (PMC9387819; doi:10.1371/journal.pone.0273237)
Supplement: S2 Table — (DOCX) [file pone.0273237.s003.docx]

**S2 Table** Genetic variants in *FGF* family members associated with height, hypertension and osteoporosis.

| Gene | Chr | SNP | Minor allele | MAF | Function | Height | | HTN | | Osteoporosis | |
| --- | --- | --- | --- | --- | --- | --- | --- | --- | --- | --- | --- |
|  |  |  |  |  |  | β ± s.e | *p*-value | OR (95% CI) | *p*-value | OR (95% CI) | *p*-value |
| *FGF2* | 4 | rs308387 | A | 0.052 | intron | 0.234 ± 0.068 | **5.85 × 10^-4^** | 1.023 (0.96-1.09) | 0.487 | 1.062 (0.94-1.20) | 0.317 |
|  | 4 | rs546560809 | G | 0.021 | intron | 0.311 ± 0.107 | **3.59 × 10^-3^** | 0.956 (0.87-1.06) | 0.38 | 1.138 (0.95-1.36) | 0.159 |
|  | 4 | rs117648266 | T | 0.054 | intron | -0.178 ± 0.067 | **7.88 × 10^-3^** | 1.033 (0.97-1.10) | 0.306 | 1.020 (0.91-1.15) | 0.743 |
|  | 4 | rs1476214 | A | 0.435 | intron | -0.071 ± 0.031 | **0.021** | 1.013 (0.98-1.04) | 0.388 | 0.959 (0.91-1.01) | 0.130 |
|  | 4 | rs150467824 | G | 0.021 | intron | -0.239 ± 0.105 | **0.023** | 0.945 (0.86-1.04) | 0.257 | 1.062 (0.89-1.27) | 0.513 |
|  | 4 | **rs167428** | C | 0.087 | intron | 0.112 ± 0.054 | **0.037** | 1.040 (0.99-1.09) | 0.122 | 1.131 (1.03-1.24) | **8.59 × 10^-3^** |
|  | 4 | rs308402 | T | 0.122 | intron | -0.041 ± 0.046 | 0.379 | 0.956 (0.92-1.00) | **0.043** | 1.043 (0.96-1.13) | 0.310 |
|  | 4 | rs308442 | A | 0.172 | intron | 0.062 ± 0.040 | 0.121 | 0.994 (0.96-1.03) | 0.748 | 1.078 (1.01-1.16) | **0.036** |
| *FGF4* | 11 | rs58166091 | A | 0.213 | - | 0.114 ± 0.037 | **2.16 × 10^-3^** | 0.989 (0.96-1.02) | 0.544 | 1.026 (0.96-1.10) | 0.436 |
|  | 11 | rs80314733 | T | 0.114 | - | 0.129 ± 0.048 | **6.78 × 10^-3^** | 0.976 (0.93-1.02) | 0.290 | 1.016 (0.93-1.11) | 0.705 |
|  | 11 | rs550530065 | A | 0.152 | - | 0.108 ± 0.042 | **0.011** | 0.972 (0.93-1.01) | 0.162 | 1.043 (0.97-1.12) | 0.275 |
|  | 11 | rs3886167 | A | 0.381 | - | 0.074 ± 0.031 | **0.018** | 0.989 (0.96-1.02) | 0.472 | 1.018 (0.97-1.08) | 0.525 |
|  | 11 | rs9666584 | A | 0.337 | intron | 0.066 ± 0.032 | **0.041** | 0.985 (0.96-1.01) | 0.304 | 1.045 (0.99-1.11) | 0.131 |
|  | 11 | rs2070678 | A | 0.227 | intron | 0.072 ± 0.036 | **0.047** | 0.989 (0.96-1.02) | 0.504 | 1.032 (0.97-1.10) | 0.335 |
|  | 11 | rs117286640 | G | 0.132 | intron | -0.002 ± 0.045 | 0.968 | 1.016 (0.98-1.06) | 0.438 | 0.910 (0.84-0.99) | **0.022** |
|  | 11 | rs117846036 | T | 0.045 | - | 0.058 ± 0.073 | 0.425 | 1.067 (1.00-1.14) | 0.060 | 0.867 (0.76-0.99) | **0.040** |
| *FGF10* | 5 | rs13154419 | G | 0.412 | intron | 0.112 ± 0.031 | **2.89 × 10^-4^** | 0.991 (0.96-1.02) | 0.545 | 0.988 (0.94-1.04) | 0.674 |
|  | 5 | rs1448039 | A | 0.500 | intron | -0.094 ± 0.030 | **1.90 × 10^-3^** | 1.006 (0.98-1.04) | 0.653 | 1.017 (0.96-1.07) | 0.528 |
|  | 5 | **rs17227836** | C | 0.054 | intron | -0.161 ± 0.067 | **0.016** | 0.993 (0.93-1.06) | 0.816 | 1.144 (1.02-1.28) | **0.021** |
|  | 5 | rs2128433 | G | 0.287 | intron | -0.079 ± 0.034 | **0.018** | 1.023 (0.99-1.06) | 0.146 | 1.008 (0.95-1.07) | 0.787 |
|  | 5 | rs138734343 | G | 0.013 | intron | 0.315 ± 0.135 | **0.020** | 1.003 (0.89-1.13) | 0.965 | 1.151 (0.92-1.44) | 0.223 |
|  | 5 | rs75784561 | G | 0.016 | intron | -0.259 ± 0.120 | **0.031** | 1.061 (0.95-1.19) | 0.291 | 0.799 (0.63-1.01) | 0.058 |
|  | 5 | rs10055386 | G | 0.391 | intron | -0.065 ± 0.031 | **0.034** | 0.987 (0.96-1.02) | 0.378 | 1.027 (0.97-1.09) | 0.336 |
| *FGF18* | 5 | **rs10463007** | T | 0.404 | - | 0.081 ± 0.031 | **9.26 × 10^-3^** | 0.990 (0.96-1.02) | 0.498 | 1.071 (1.01-1.13) | **0.014** |
|  | 5 | rs147685475 | T | 0.044 | intron | 0.178 ± 0.073 | **0.015** | 0.991 (0.93-1.06) | 0.797 | 1.054 (0.93-1.20) | 0.412 |
|  | 5 | rs734840 | T | 0.345 | - | 0.066 ± 0.032 | **0.037** | 0.996 (0.97-1.03) | 0.798 | 1.020 (0.96-1.08) | 0.498 |
|  | 5 | rs80105845 | G | 0.029 | intron | -0.123 ± 0.090 | 0.175 | 1.103 (1.02-1.20) | **0.02** | 0.943 (0.80-1.11) | 0.479 |
|  | 5 | rs74838398 | C | 0.051 | intron | -0.093 ± 0.069 | 0.179 | 1.071 (1.01-1.14) | **0.035** | 0.976 (0.86-1.10) | 0.701 |
|  | 5 | rs79711557 | T | 0.154 | intron | -0.032 ± 0.042 | 0.449 | 1.041 (1.00-1.08) | **0.041** | 1.015 (0.94-1.09) | 0.689 |
|  | 5 | rs62383987 | A | 0.275 | intron | -0.007 ± 0.034 | 0.832 | 0.991 (0.96-1.02) | 0.556 | 0.923 (0.87-0.98) | **0.010** |
|  | 5 | rs3884606 | G | 0.454 | intron | 0.039 ± 0.031 | 0.204 | 0.998 (0.97-1.03) | 0.861 | 1.073 (1.02-1.13) | **0.011** |
|  | 5 | rs10077440 | C | 0.399 | intron | -0.034 ± 0.031 | 0.278 | 0.984 (0.96-1.01) | 0.258 | 0.932 (0.88-0.99) | **0.013** |
|  | 5 | rs4559013 | A | 0.457 | intron | 0.055 ± 0.031 | 0.069 | 0.992 (0.96-1.02) | 0.576 | 1.069 (1.01-1.13) | **0.016** |
|  | 5 | rs12521055 | T | 0.333 | intron | 0.037 ± 0.032 | 0.257 | 0.982 (0.95-1.01) | 0.229 | 1.071 (1.01-1.13) | **0.018** |
|  | 5 | rs78993631 | G | 0.280 | intron | -0.012 ± 0.034 | 0.722 | 0.988 (0.96-1.02) | 0.442 | 0.933 (0.88-0.99) | **0.026** |
|  | 5 | rs9790978 | T | 0.126 | intron | -0.056 ± 0.046 | 0.226 | 0.991 (0.95-1.03) | 0.668 | 0.917 (0.84-1.00) | **0.043** |
| *FGF22* | 19 | rs62134260 | G | 0.047 | upstream | -0.212 ± 0.071 | **2.97 × 10^-3^** | 0.999 (0.94-1.07) | 0.975 | 0.999 (0.88-1.14) | 0.989 |
|  | 19 | rs7246140 | T | 0.309 | intron | -0.087 ± 0.033 | **7.93 × 10^-3^** | 0.988 (0.96-1.02) | 0.437 | 0.979 (0.92-1.04) | 0.473 |
|  | 19 | rs56704509 | A | 0.030 | intron | -0.215 ± 0.089 | **0.016** | 0.929 (0.86-1.01) | 0.080 | 0.960 (0.82-1.13) | 0.625 |
|  | 19 | **rs8109113** | G | 0.024 | intron | -0.241 ± 0.100 | **0.016** | 0.901(0.82-0.99) | **0.028** | 1.021 (0.85-1.22) | 0.824 |
|  | 19 | rs11572906 | T | 0.023 | downstream | -0.214 ± 0.101 | **0.033** | 0.946 (0.86-1.04) | 0.244 | 0.962 (0.80-1.16) | 0.680 |
|  | 19 | rs8102093 | C | 0.232 | - | 0.055 ± 0.036 | 0.126 | 0.990 (0.96-1.02) | 0.569 | 0.926 (0.87-0.99) | **0.020** |

Age, sex and body mass index (BMI) were included as covariants in all genetic models. SNPs associated with both hypertension and osteoporosis in common and had *P* < 0.05 are indicated in bold. The *p*-values which are satisfied the Bonferroni-corrected significance level regarding each gene are indicated in bold and underlined. Abbreviations: SNP, single nucleotide polymorphism; Chr, chromosome; MAF, minor allele frequency; HTN, hypertension; β, regression coefficient; s.e, standard error; OR, odd ratio; CI, confidence interval.
