# Supplementary material for: FGFRL1 and FGF genes are associated with height, hypertension, and osteoporosis
Source: PLoS One. 2022 Aug 18;17(8):e0273237. doi: 10.1371/journal.pone.0273237 (PMC9387819; doi:10.1371/journal.pone.0273237)
Supplement: S3 Table — Abbreviations: A1, minor allele; A2, major allele. The lower the Regulome DB score, the greater the effect on SNP. (DOCX) [file pone.0273237.s004.docx]

**S3 Table** HaploReg results of genetic variants in *FGFRL1* that were associated with both hypertension and osteoporosis. Abbreviations: A1, minor allele; A2, major allele. The lower the Regulome DB score, the greater the effect on SNP.

| **SNP** | **A1** | **A2** | **HaploReg** | | | | | **RegulomeDB** |
| --- | --- | --- | --- | --- | --- | --- | --- | --- |
|  |  |  | **Promotor histone marks** | **Enhancer histone marks** | **DNase** | **Proteins bound** | **Motifs changed** |  |
| rs13143527 | A | G | 18 tissues | 12 tissues | - | - | BDP1,Ets | 4 |
| rs55639339 | T | C | BRN | 10 tissues | BLD | - | BDP1, Evi-1, NRSF | 4 |
